# Can written prompts help medical residents to accurately monitor their own communication skills and those of others?

**DOI:** 10.1007/s10459-024-10364-w

**Published:** 2024-09-05

**Authors:** F. O. de Meijer, N. Nyamu, A. B. H. de Bruin

**Affiliations:** 1 Department of Postgraduate Medical Education, Aga Khan University, Nairobi, Kenya; 2 School of Health Professions Education, Maastricht University, Maastricht, The Netherlands; 3 Deparment of Family Medicine, Aga Khan University, Nairobi, Kenya; 4 Faculty of Health, Medicine and Life Sciences, Maastricht University, Maastricht, The Netherlands

**Keywords:** Communication skills training, OSCE, Monitoring accuracy, Cue diagnosticity, Judgments of satisfaction, End of life care

## Abstract

In healthcare, effective communication in complex situations such as end of life conversations is critical for delivering high quality care. Whether residents learn from communication training with actors depends on whether they are able to select appropriate information or ‘predictive cues’ from that learning situation that accurately reflect their or their peers’ performance and whether they use those cues for ensuing judgement. This study aimed to explore whether prompts can help medical residents improving use of predictive cues and judgement of communication skills. First and third year Kenyan residents (N = 41) from 8 different specialties were randomly assigned to one of two experimental groups during a mock OSCE assessing advanced communication skills. Residents in the intervention arm received paper predictive cue prompts while residents in the control arm received paper regular prompts for self-judgement. In a pre- and post- test, residents’ use of predictive cues and the appropriateness of peer-judgements were evaluated against a pre-rated video of another resident. The intervention improved both the use of predictive cues in self-judgement and peer-judgement. Ensuing accuracy of peer-judgements in the pre- to post-test only partly improved: no effect from the intervention was found on overall appropriateness of judgements. However, when analyzing participants’ completeness of judgements over the various themes within the consultation, a reduction in inappropriate judgments scores was seen in the intervention group. In conclusion, predictive cue prompts can help learners to concentrate on relevant cues when evaluating communication skills and partly improve monitoring accuracy. Future research should focus on offering prompts more frequently to evaluate whether this increases the effect on monitoring accuracy in communication skills.

## Introduction

In healthcare, effective communication by healthcare providers is critical for delivering high quality care. This is especially true in complex clinical situations, such as breaking bad news to terminally ill patients or end-of life discussions. Future specialists, therefore, need to acquire appropriate verbal and non-verbal skills to effectively handle difficult conversations. Training with actors can provide good experiences to help residents (specialists in training) decide which aspects of their performance need further attention (Maguire & Pitceathly, 2002). Yet, this can only be effective when they are able to accurately monitor their performance (Zimmerman & Schunk, 2011).

### Cue-diagnosticity and monitoring accuracy

**Fig. 1:**
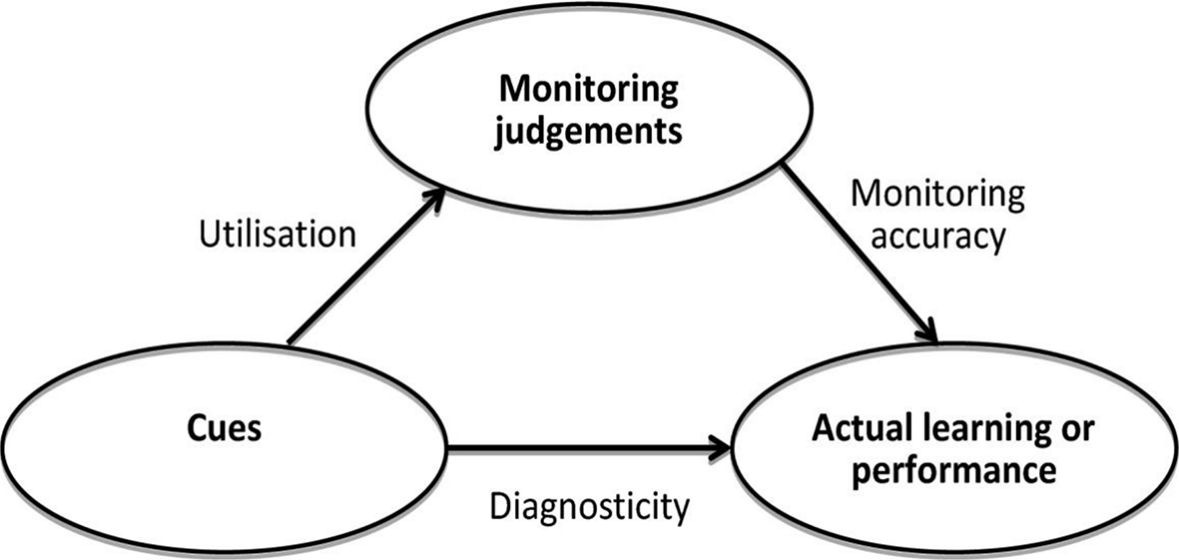
Conceptual framework relating monitoring accuracy, cue-utilization and cue-diagnostcity (de Bruin & van Merrienboer, 2017)

Monitoring performance is part of the self-regulated learning cycle. This cycle is comprised of planning for a learning task, monitoring performance and reflection on the outcomes and setting of learning goals (De Bruin & van Merrienboer, 2017). The accuracy of self-monitoring depends on the information or ‘cues’ one selects from a given situation and whether these cues accurately mirror performance, a term called cue-diagnosticity. It is important that learners focus on relevant, also called predictive, cues and use these as a basis for their judgements while ignoring irrelevant cues. This process, called cue-utilization, ensures ensuing monitoring judgments are accurate, see Fig. 1 (Koriat, 1997; de Bruin et al., 2017). In the context of breaking bad news for example, signals that the patient did not sufficiently understand the severity of his/her disease may cause a doctor-in-training to adopt a different strategy and more clearly explain consequences of the disease during a next attempt Table 1.

**Table 1:** Table of terms

Cues: signals or hints one can percept in a given situation
Cue-utilization: the process in which one consciously and unconsciously selects different types of cues for ensuing judgment
Judgments of Satisfaction (JOS): judgments formed by processing different types of cues that the situation emits
Predictive cues: cues that accurately mirror performance
Predictive prompts: instructive prompts that guide learners to select predictive cues for their judgments

Unfortunately, learners often rely on non-predictive (irrelevant) cues when evaluating their performance such as familiarity with the task or time taken to complete the task. These cues are often not related with learners’ actual performance (Thiede & Anderson, 2003). This could lead to a monitoring judgement that is too positive with subsequent inadequate formulation of learning goals and little improvement of communication skills. On the other hand, a judgment that is too negative may lead to lack of confidence and subsequent avoidance of difficult conversations (Cavalcanti & Sibbald, 2014; Dunlosky & Rawson, 2012).

### Judgements of satisfaction type a, b and c

Although in many learning situations it is not exactly known which cues are more predictive for good performance than others, Wagner-Menghin et al. (2016) studied cue-diagnosticity in the context of communication skills. They categorized students’ self-evaluations of their video-recorded communication skills, so called ‘Judgments of Satisfaction (JOSs), in 3 different types based on Koriat’s extensively studied cue-utilization framework (Koriat, 1997):

JOS type (a): completeness (or lack thereof) of covered content. When students form an a-type judgement, they are satisfied when they cover all items of a conversation that were explained as important during their training. Dissatisfaction occurs when certain topics are not covered (Wagner-Menghin, et al., 2016). In a breaking bad news conversation with a patient, for example, a JOS type (a) judgement could be ‘satisfied with the fact that I explained localization of the tumor’;

JOS type (b): quality of performing a process skill. When forming a b-type JOS, students are satisfied when they are able to apply communication techniques in the way they were taught during their training course. When they are not able to observe the application of these process skills, it leaves them dissatisfied. A JOS type (b) judgement would for example be: ‘satisfied that I avoided jargon when explaining localization of the tumor’;

JOS type (c): appropriateness of a process skill to reach goal. Students who use type c judgements are able to include reflections on how the content of the discussion and the employed communication skills affect the patient. For example, ‘satisfied as the patient nodded and correctly repeated what I had said, showing they understood the explanation of the tumor localization well’;

Whereas low to moderate performers mostly focused on the content of the conversation and the communication-techniques used (type a and b) by the student, high-performers paid more attention to the responsiveness of patients to the communication skills used (type c). It seems, that students who use only JOS type a or JOS type b, are still unduly concerned with coverage of content or application of skills and are not able to prioritize what is important in order to reach their goal. Good communicators evaluate whether their communication techniques have the intended effect on the patient by paying attention to the patient’s verbal and non-verbal responsiveness, a predictive cue in this context (Borg, 2010).

#### Self-generation of cues and prompting

Self-generation of cues is known to improve monitoring accuracy, especially if it is combined with trainer’s feedback (Brydges et al., 2012; Hammoud et al., 2012). The combination of self-generated feedback and teacher’s feedback is used for teaching communication skills in many postgraduate training models nowadays. Often, residents are asked to self-assess one of their real- life videoed consultations and to share this reflective self-assessment with a (trained) expert for their feedback (Wouda, & van de Wiel, 2014). The self generation of feedback is thought to improve retention in long term memory due to the mental effort it takes for a learner to come up with these cues. Ensuing feedback from a trainer is also needed to ensure feedback is accurate. This process can potentially be made even more efficient by guiding or prompting the resident during the self-assesment. Thiede et al., 2005, showed that students who were asked to produce 5 key words that captured the essence of a text, showed better monitoring accuracy then students who were not given this prompt. As such, prompting provides an opportunity to reduce cognitive demands and prevents learners from wasting time and energy on irrelevant cues (Brouwers et al., 2017; De Bruin & van Merrienboer, 2017). In conclusion, prompting learners to self-generate cues from a learning situation could lead to focusing on predictive cues resulting in adequate judgments and formulation of learning goals.

Although we know that reflective practice in communication skills training improves learning, we do not know much about the usefulness of prompting learners in this context (Hawkins, Osborne, Schofield, Pournaras, & Chester, 2012; Karnieli-Miller et al., 2018). Moreover, as current research on cue-utilization solely originates from the global North, there is need for literature from the global South to add to the applicability of cue utilization theory across different cultural and educational settings. In this study, we use the judgement of satisfaction classification type c identified by Wagner-Menghin et al. (2016) to study if guiding learners with self-generative cue prompts indeed improves the use of predictive cues when monitoring communication skills. This could potentially help future specialists in better self-monitoring and subsequent acquisition of communication skills.

Our first hypothesis is that trainees exposed to self- generative cue prompts demonstrate improved use of predictive JOS Type c cues in self- and peer-judgement. Our second hypothesis is that trainees exposed to self-generative cue prompts show improved accuracy of peer- judgements. This study therefore sought to determine whether (a) prompting residents with written cue prompts after a MOCK common OSCE training improved their use of predictive cues on subsequent self- and peer evaluations of communication skills. And whether (b) this subsequently lead to improved appropriateness or accuracy of peer- judgements.

## Methods

### Study design

This was an experimental double blind control group design with both an intervention and a control arm. A pre- and post test was done to evaluate the effect of the intervention.

### Participants and study context

This study was conducted amongst a cohort of first- and third-year residents from multiple medical specialisms at the Aga Khan University Hospital, Nairobi, Kenya. As part of their four-year master program, residents in both year 1 and 3 learn from communication experts how to optimize their communication skills in complex situations. These training sessions take place monthly and participating residents are from 8 different specialties (Pediatrics, Surgery, Internal Medicine, Obstetrics-Gynecology, Family Medicine, Clinical Pathology, Radiology and Anesthesiology). This cross- specialty set up was installed to ensure communication training is prioritized in all medical specialties and to enhance communication and teamwork between medical specialties. At the end of their 1st and 3rd year, residents undergo a mock OSCE with actors before they are assessed during a communication OSCE. During the mock OSCE, residents rotate through 5 different scenarios assessing communication skills in the following situations: breaking bad news, conflicts with colleagues, end of life discussions, admitting medical errors and non-adherence to treatment. Each station has 1 experienced examiner and 1 actor. The examiners had access to an assessment rubric for each of the topics during the MOCK OSCE. The residents were informed about the topics of the stations before hand. Examples of assessment rubrics for the topics had been given during monthly trainings. Also these rubrics, used in previous years, were available on the online learning platform. For the MOCK OSCE, the assessment rubrics were not made available for residents as it was thought this might have potentially directed residents to use JOS a, b or C. Before entering the station the students read the scenario of the case during 2 min and were asked to address the concerns of the patient. All the 59 residents participating in the mock OSCE were invited to participate in the study of which 48 consented to participate (81%). However, seven residents could not make it for the post-test because of clinical duties/emergencies, so finally 41 residents participated in the study. Residents were randomly assigned to the groups as shown in Table 2.

**Table 2:** Numbers and percentages of residents across intervention groups

Variables	Intervention		Control	
	*N*	%	*N*	%
*Gender*				
Female	8	40	12	57
Male	12	60	9	43
*Years of Residency*				
1	11	55	10	48
3	9	45	11	52
*Specialty*				
Pathology	2	10	2	10
Radiology	3	15	3	14
Anesthesiology	1	5	2	10
Surgery	2	10	3	14
Obstetrics/Gynaecology	4	20	3	14
Paediatrics	2	10	4	19
Family medicine	3	15	1	5
Internal medicine	3	15	3	14
Total	20		21	

### Procedure

**Fig. 2:**
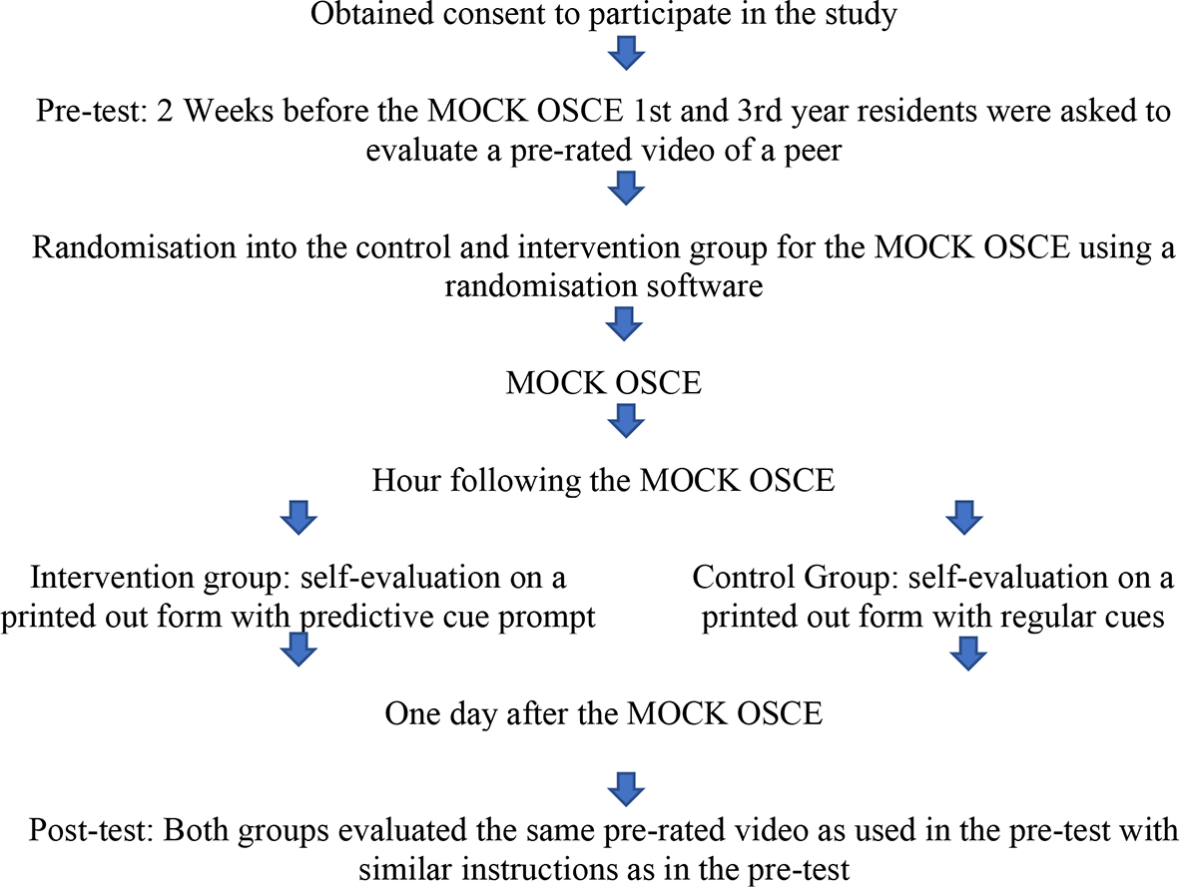
Flow–diagram of the study procedures

The procedures for the study are illustrated in Fig. 2: during a regular communication training 2 weeks before the mock OSCE, 1st and 3rd year residents were asked to evaluate a pre-rated video that served as a pre-intervention test. The 10-minute video was made of one of the alumni during the mock OSCE training in previous years in order to reduce differences between the pre/post test and the intervention as much as possible. The video was shown with the alumni’s consent. The video was shown twice and the following instructions were given:

- While watching the *first time*, please note down passages/ bits of text in which you clearly feel **satisfied** with the candidate’s **communication techniques** as well as scenes in which you feel **dissatisfied** with the candidate’s **communication techniques.**
- When watching the video for the *second time*, briefly describe **what is happening in the selected passages** and explain **WHY you are satisfied/not satisfied with the communication skills** of the candidate in this passage.

- Passage 1: what is happening? Why satisfied/dissatisfied?
- Passage 2: Etc

During the Mock common OSCE training, residents were randomly divided into 2 arms by randomization software ensuring random but equal distribution of gender and type of residency amongst groups. The residents and instructors did not know whether they were placed in the intervention or the control group. Directly after each station, residents received brief verbal group-feedback from examiners. In the hour following the MOCK OSCE, residents were asked to write down their self-evaluation for each of the five stations based on the written prompt given by a research assistant. Residents in the intervention group received paper prompts steering them to use predictive cues in their self-evaluations. Residents in the control group received regular written questions for their self-evaluations. One day after the mock OSCE, residents were asked to evaluate the same pre-rated video as used in the pre-test with similar instructions to the pre-test.

### Materials

#### The intervention arm

we created a self-generative, written cue prompt based on the notion that JOS type c cues are most predictive for performance. The cue prompt was formulated as follows.

- Can you give examples of moments where your communication skills have the intended effect on the patient/colleague? What went well? How is this confirmed in verbal communication or body language by the patient? (eye contact, gestures, body movement, posture, facial expressions, repetitive movements of the extremities, vocalizations)?
- Can you give examples of moments where your communication skills have no or an unintended effect? Why is this so? What could you improve?

Control arm: Residents who were allocated to the control group receiving regular cues were asked to self-evaluate their performance. The regular questions were formulated as follows:

- Can you give examples of moments where you are satisfied with your communication skills? What went well?
- Can you give examples of moments where you were not satisfied with your communication skills? Why is this so? What could you improve?

#### Pre- and post-test

A 10-minute video of a complex (simulated) end of life conversation was chosen as a pre- and post-test and was pre-rated by 4 communication experts involved in communication training (1 family physician, 1 pediatrician, 2 palliative care doctors). The performance of the doctor in the video was mostly erroneous and divided into categories based on the PREPARED model, a widely used acronym in end of life discussions (Clayton et al., 2007). For each category, experts independently classified the performance in the video as satisfactory or dissatisfactory. They unanimously agreed on satisfactory performance in 1 category (Relate to the Person) and dissatisfactory performance in 5 categories (Prepare for the discussion; Explore priorities and concerns; Provide information; foster Realistic hope and Encourage questions and Document). After discussion, the 7th category (Acknowledging emotions and concerns) was split into a satisfactory part and a non-satisfactory part. The process resulted in a standard of 8 categories against which the residents’ judgments in the pre-and post-test could be scored on appropriateness and completeness, both criteria for monitoring accuracy, see Table 3.

**Table 3:** Pre- and post-test: 8 categories of (dis)satisfaction pre-rated by experts based on the PREPARED model (Clayton et al., 2007)

Classification	Description of theme
Satisfactory performance	*Relate to the person*: describing appropriate initial part of the introduction by the doctor and making rapport with the relative ***A****cknowledge emotions and concerns*: describing doctor’s ability to maintain calmness and show concern despite relative’s anger
Unsatisfactory performance	***P****repare for the Discussion*: describing doctor’s inability to install confidence in relative that he works as part of a team and would be able to address the situation
	***E****xplore priorities and concerns*: describing ineffective exploration of the relative’s perceptions & concerns with regards to advanced directives and end of life care
	***P****rovide information*: describing inability to clearly and empathically discuss end of life care, goals & advanced directives with relative
	***A****cknowledging emotions and concerns*: describing ineffective way of addressing relative’s anger
	*Foster****R****ealistic hope*: describing ineffective way of focusing on what CAN be done at the end of life with regards to symptom control
	***E****ncourage questions &**** D****ocument*: describing ineffective way of trying to involve other family members and the ethics committee in order to reach a consensus

### Analysis

#### Qualitative steps of analysis

First, inductive category application was performed by dividing residents’ written communication judgments of a peer’s communication skills (during the pre- and post-test) into categories, namely appropriateness of the judgements according to the pre-set ‘standard’ by 4 experienced faculty members. This was done by the author and checked by a colleague. Then, self-judgements made after the mock OSCE were classified into ‘types of cues used’ as presented in the Wagner-Menghin model: type a, type b or type c (predictive type). This was independently done by the author and a colleague and compared. Instructors during the MOCK OSCE didn’t know which group students were in. Also, the author and colleague did not know in which group (intervention versus control) the judgements had been made. Cues which were classified differently were discussed and an agreement was reached on how to classify the items. The same was done for cues during the pre- and post-test.

#### Quantitative steps of analysis

We counted frequency of JOS types a, b and c cues used during the Mock OSCE (intervention) and during the pre and posttest. One-way MANOVA was first used to evaluate differences in JOS use between the intervention and control groups in the hour following the MOCK OSCE. Subsequently, 2 × 2 repeated-measures ANOVA with time (pre/post) as the within subjects independent variable and group (control/intervention) as the between-subjects variable, was used to calculate differences in JOS-use and accuracy of judgments. Excel was used to manage the category application and scores and to derive the JOS A, B and C frequency counts. JASP was used for statistical analysis.

#### Inductive category application

To evaluate the completeness of the peer-judgements per resident, appropriate judgements during the pre- and post test were scored across the range of the 8 categories. For each appropriately judged category, 1 mark was assigned, irrespective of the number/type of judgements made within that category. This means that a total of 8 marks per resident on the evaluation of the video could be obtained both pre and post intervention. The same was done for inappropriate judgements.

## Results

### Recognition of JOS c predictive cues in self-assessment after the mock OSCE

**Fig. 3:**
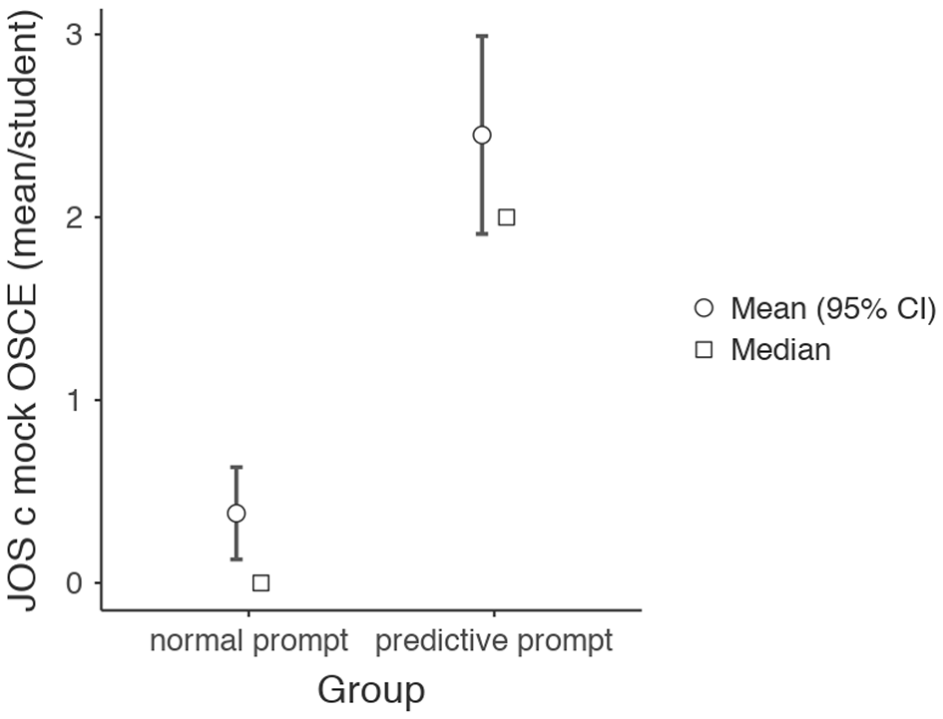
Mean JOS c use per student for self-judgment after the MOCK OSCE. *Note* A mean of 2.45 (SD 1.23) JOS c type observations were made per student in the predictive prompt group while 0.381 (SD 0.590) were made in the regular prompt group

When self-assessing in the hour following the mock exercise, residents in the intervention group were significantly better able to use JOS-c judgments for self-assessment (49 out of 72 judgments = 68%) than residents in the control group (8 out of 88 = 9%), *F*(1,39) = 47.6, *MSE* = 43.9, *p* <.01. JOS a and JOS b judgments were significantly more used in the control group, *F*(1,39) = 12.8, *MSE* = 23.4, *p* <.01 and *F*(1,39) = 8.0, *MSE* = 19.7, *p* =.007 respectively, see Table 4. Mean JOS c use per resident for the intervention and control group is illustrated in Fig. 3.

**Table 4:** Type of judgement used in self-assessment after mock OSCE across intervention groups

Type of JOS	Example	Frequency *N* (%)	
		Predictive prompt	Regular prompt
JOS a: completeness (or lack thereof) of covered content	“I did not address the issue of my fellow resident’s demeanor to the assistant, calling her ‘useless’. I should have found a way to let him know that even if agitated, his reaction should still be within bounds of respect”, (p. 54)	5 (7)	33 (38)
JOS b: quality of performing a process skill	“After breaking bad news, I did not pause intentionally to see whether the patient was following, I talked too long before giving the patient time to internalize; at one point it sounded like a monologue”, (P. 2)	18 (25)	47 (53)
JOS c: appropriateness of a process skill to reach goal	“I was clear and firm when stating that it was unacceptable. without threatening. The recipient did not feel like she was under attack. Hence more responsive to what I was saying”, (p.53)	49 (68)	8 (9)

### Pre and posttest comparison evaluating peer- assessment across intervention groups

A total of 234 cues were used pre-intervention and 231 post intervention with a mean number of 5.6 cues per resident during both the pre and posttest.

#### JOS Type

**Fig. 4:**
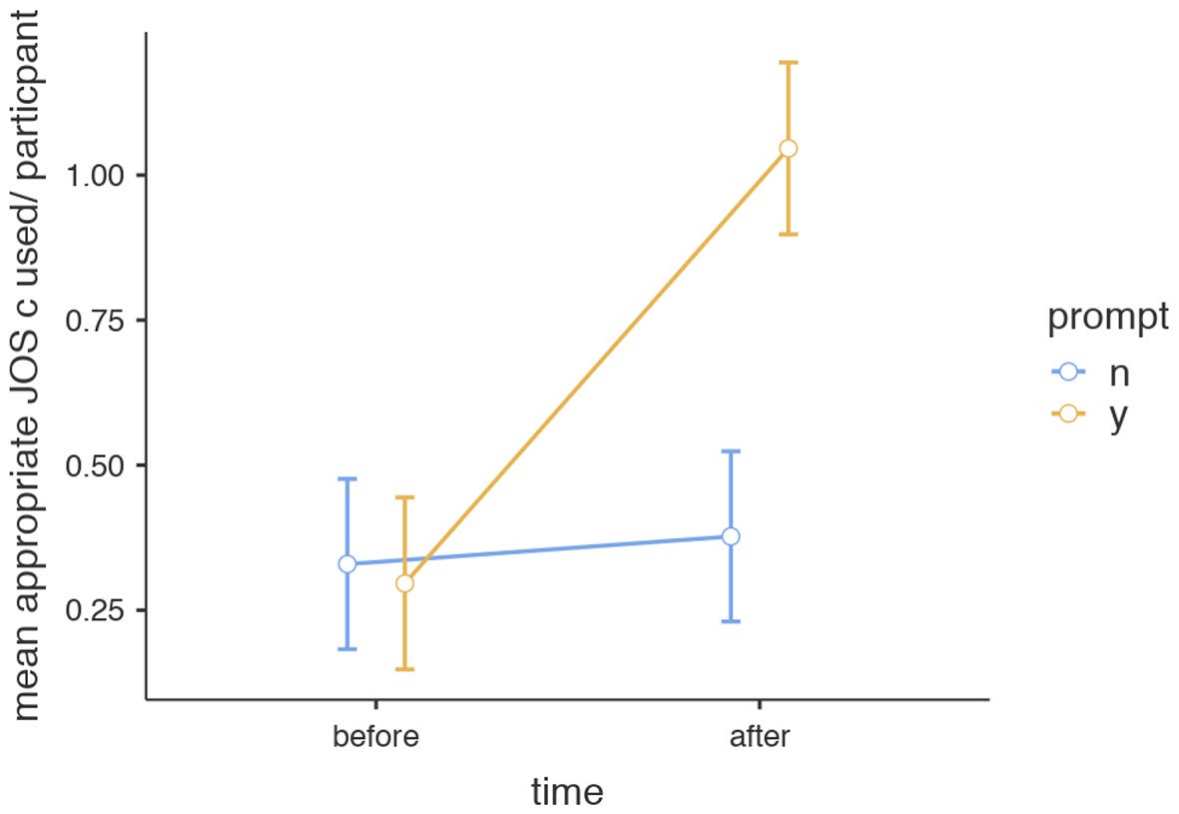
Mean use of predictive JOS c cues (and standard errors) on pre- and posttest of a video on peer-judgement across intervention groups. *Note* n = no, did not receive predictive prompt, y = yes, received predictive prompt

First, judgements were classified according to appropriateness against the pre-rated standard of the peer resident in the video and then categorized in types of judgements, namely type a, type b and type c (= predictive type). The results of the repeated-measures ANOVA showed no effect of the intervention on appropriate JOS a use *F*(1,39) = 1.29, *MSE* = 0.87, *p* =.26. Nevertheless, a crossover interaction was seen in the *in*appropriate use of JOS a cues *F*(1,39) = 5.38, MSE = 10.24, *p =*.03, indicating the effect of the intervention on the use of inappropriate JOS a was opposite to that of the control group: where the control group used more inappropriate JOS a from pre to post test, this effect was reversed for the intervention group. No effect of the intervention was found on JOS b use and total number of appropriate or inappropriate judgements. However, there was a significant main effect of the intervention on the average number of JOS c (predictive) cues used *F*(1,39) = 8.86, *MSE* = 3.26, *p* =.005, *η*^*2*^ = 0.08 and a significant interaction effect between time and intervention group *F*(1,39) = 6.87, *MSE* = 2.53, *p* =.01, *η*^*2*^ *=.*06. Post hoc comparisons using the Tukey t-test indicated a significant increase in appropriate JOS c cues within the intervention group from pre (*M* = 0.30, *SD* = 0.47) to post test (*M* = 1.05, *SD* = 0.94), *p* =.002, see Fig. 4. In the control group, no significant difference from pre (*M* = 0.33, *SD* = 0.59) to post test (*M* = 0.38, *SD* = 0.60) was found, *p* =.99. Mean JOS c use on the post test was significantly higher in the intervention group (*M* = 1.05, *S*D = 0.94) when compared with the control group (*M* = 0.38, *SD* = 0.60), *p* =.01). No difference in appropriate JOS c use was found in the pre-test between intervention group (*M* = 0.30, *SD* = 0.47) and control (*M* = 0.33, *SD* = 0.59), *p* =.99. JOS c cues almost always led to appropriate judgements (98%) and only once led to an inappropriate judgement.

#### Completeness of judgements of a peer-resident across the pre-rated themes

Then, peer-judgements (appropriately satisfied, appropriately dissatisfied, inappropriately satisfied and inappropriately dissatisfied) were scored across the pre-rated themes to evaluate completeness of peer communication in the video, see Table 5 for example quotes in the theme ‘explore priorities and concerns’.

**Table 5:** Example quotes per JOS-type for the theme ‘explore priorities and concerns’

Type of JOS	Appropriateness	Example quotes
JOS a	Appropriately dissatisfied	*“Doctor explaining about the ventilation. He did not introduce the procedure and did not seek to identify what was the clients’ prior knowledge with regard to the same.”* (p1)
	Inappropriately satisfied	*“Trying to understand the relative’s concerns. Satisfied - asked the relative to explain what is happening despite knowing already - no room for miscommunication.”* (p4)
JOS b	Appropriately dissatisfied	“*Generally, doctor is asking closed ended questions. The best would-be open-ended questions which allow the patient to open up.”* (p28)
	Inappropriately satisfied	*“The caretaker reports his frustrations and resident listens and tries to address them in the same time maintaining his position. This is good communication to listen to patient and show empathy at the same time using the same direction of conversation to point out his communication.”* (p18)
JOS c	Appropriately dissatisfied	*“How do you feel about it. He wanted an update on his sentiments on the matter. Dissatisfied since the son was clearly distressed and this aggravated him even more. He should have phrased it differently.”* (p32)
	Inappropriately satisfied	*None*

**Fig. 5:**
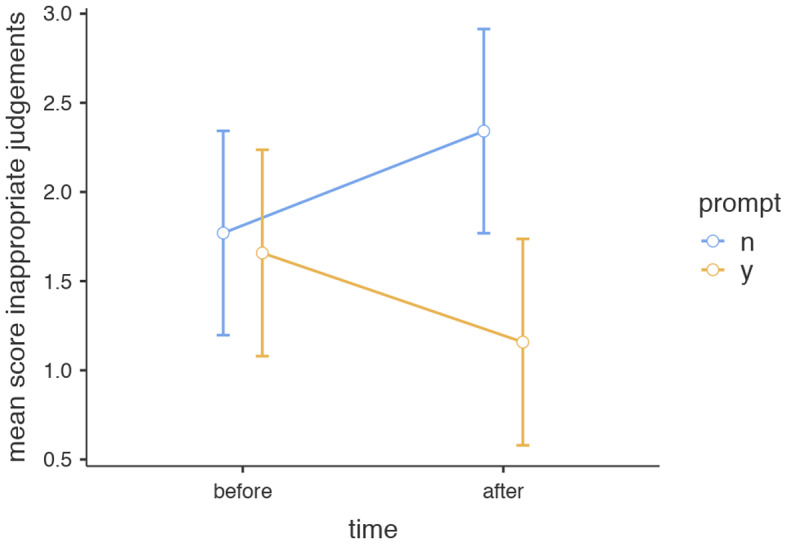
Completeness scores (and standard error of the mean scores) of Inappropriate judgments on pre- and posttest across intervention groups. *Note* n = no, did not receive predictive prompt, y = yes, received predictive prompt

A cross over interaction was seen in the total appropriate group, *F*(1,39) = 4.33, *MSE* = 5.78, *p* =.04, *η*^*2*^ = 0.03, indicating the effect of the intervention on the completeness of appropriate judgements was opposite to that of the control group: where the completeness scores in the intervention group improved from pre to post test, this effect was reversed for the control group. Another cross over interaction was seen in the inappropriately satisfied group, *F*(1,39) = 4.34, *MSE* = 14.8, *p* =.04, *η*^*2*^ = 0.04, indicating that where completeness scores for inappropriate judgments decreased for the intervention group, they increased for the control group. Nevertheless, no overall differences in scores were noted between these groups on a post hoc test. Scores in the total inappropriately judged themes decreased significantly in the intervention group when compared with the pre-test *F*(1,39) = 4.38, *MSE* = 8.59, *p =*.04, *η*^*2*^ = 0.06 which is considered a small to medium effect size, see Fig. 5. No overall differences were seen in appropriately satisfied, appropriately dissatisfied and inappropriately dissatisfied judgments, see Table 6.

**Table 6:** Descriptive statistics for completeness of judgements on pre and post tests across intervention groups

prompt	Before		After	
	Mean score (%)	SE	Mean score (%)	SE
*Appropriately satisfied*				
N	28.4	7.84	26.0	7.84
Y	34.8	7.89	47.3	7.89
*Appropriately dissatisfied*				
N	43.7	5.18	31.8	5.18
Y	31.7	5.22	32.6	5.22
*Total Appropriate*				
N	3.19	0.337	2.43	0.337
Y	2.60	0.340	2.90	0.340
*Inappropriately satisfied*				
N	27.1	4.84	36.6	4.84
Y	27.6	4.88	20.1	4.88
*Inappropriately dissatisfied*				
N	7.19	2.63	2.42	2.63
Y	0.04	2.66	2.54	2.66
Total inappropriate *				
N	1.77	0.290	2.24	0.290
Y	1.66	0.293	1.26	0.293

*Note* * *p* <.05

## Discussion

To our knowledge, this study is the first experimental double blinded control design study to evaluate whether predictive communication prompts improve cue-utilization and accuracy of monitoring judgements. Moreover, this is the first study from the Global South to evaluate cue-utilization. The use of predictive cues in self- and peer-assessment were evaluated after a MOCK OSCE assessing communication skills. And the appropriateness of judgements of satisfaction when assessing a peer were evaluated against a pre-rated video and compared before and after the intervention and across groups. When evaluating the effect of communication training in health professionals, it is important to not only focus on performance but to understand the process through which learning occurs. This can be done through the cue-utilization model (De Bruin et al., 2017). However, quantifying cue-use and monitoring judgements is complex when evaluating communication skills as evaluation is often qualitative and comparisons between different consultations are difficult to make. By using a pre-rated communication video, we were able to ‘rate’ cue utilization and monitoring judgments before and after a training intervention.

### Cue utilization

The findings of this study confirmed our first hypothesis that trainees exposed to self generative predictive prompts demonstrate improved use of predictive cues when evaluating both their own and other’s communication skills. Our study showed that offering residents predictive cue prompts, stimulating them to evaluate a patient’s responsiveness to communication techniques used by the doctor (JOS type c) rather than focusing on less important cues such as only the content of the discussion (JOS type a) or the communication techniques alone (JOS type b), led to an increase in predictive cue-use post-intervention. Although the overall number of JOS c used post intervention in our study was relatively small, the intervention had both an effect on self-judgement and judgements about others. In contrast to the study by Wagner-Menghin et al. (2016), in our study, we asked doctors to craft written judgements which is more effortful than scoring or giving verbal judgements. The result may have been a smaller number of cues than would be produced if they were speaking. Nevertheless, written judgements may better facilitate long-term retention of learning (Son, & Simon, 2012). More research is needed to evaluate whether written self-evaluation has a stronger effect on monitoring accuracy then verbal evaluation. Moreover, more frequent trainings might be needed to cause the desired effect post-intervention: similar to the ‘spacing effect’, several trainings over time might benefit long-term retention of predictive cues and facilitate recall during OSCEs and real-life situations (Son, & Simon, 2012). Simulation training with actors forms an excellent opportunity to offer residents predictive cue prompts, as this can act as a mental try out leading to improved reliance on these cues. This may lead a resident to also anticipate on these types of cues during consultations in real life.

Interestingly, while the use of inappropriate JOS a decreased in the intervention group as expected, there was a contrary effect in the control group: residents in this group tended to focus slightly more on JOS a cues after being given the normal prompt and were more often inappropriately satisfied on the post-test, see example given in Table 4. Possibly, the training made participants in the control group aware of certain content aspects that had to be discussed during end of life discussions and as a result they were satisfied when those content aspects were ‘ticked’ during the post-test, even when this was not done effectively. In conclusion, a regular prompt asking residents to indicate moments when they were satisfied with their communication skills does not seem sufficient to reach accurate judgments.

### Monitoring accuracy

Our study showed that JOS c cues predominantly led to appropriate judgements (98%) with only 1 exception in our study. This further builds on the results reported by Wagner-Menghin et al. (2016) where JOS c cues were found to be related to improved performance by undergraduate students. Apparently, evaluating whether the intended effect on a patient is reached, hardly ever leads to inaccurate satisfaction or inaccurate dissatisfaction. This in turn can lead to improved performance. If a doctor however overlooks to pay attention to a patient’s body language or verbal response, he/she might be inaccurately satisfied with the way communication techniques were implemented with inadequate formulation of learning goals and decreased performance as a consequence. In short, focusing on JOS c type cues avoids making this mistake.

To evaluate whether the intervention in our study led to improved monitoring accuracy, we evaluated the effect on the overall number of appropriate and inappropriate judgements as well as on the completeness of these judgements. Our study only partly confirmed our second hypothesis that trainees exposed to self-generative cue prompts show improved accuracy of judgements: There was no overall improvement in appropriate and/or inappropriate judgements. This is most likely due to the relatively low number of JOS c cues used. Our second hypothesis was partially confirmed as a reduction in inappropriate judgements across the various themes was noticed in the intervention group: besides evaluating the number of appropriate and inappropriate judgments, it is important to evaluate completeness of the judgements made. This gives us more insight into their quality and whether residents are able to make judgements on all important aspects related to end of life discussions. Overall, judgements made by residents in our study were narrow, based on less then 40% of possible themes. This might have been due to prior teaching or due to feedback after the MOCK OSCE. Diagnostic prompts did not significantly improve this score in appropriate judgements. They did however decrease the score of inappropriate judgements made. This means that inappropriate judgments are made based on significantly fewer themes due to the intervention.

### Implications for complex communication skills training

Post graduate medical training programmes should consider the incorporation of predictive prompts in communication skills training as these may provide trainees with an opportunity to develop both their communication and self-assessment skills.

The FEEDBK model, a novel feedback tool used in communication training that encompasses developing learning tasks after an encounter with a patient, is an example of a model that could benefit from use of predictive prompts. Incorporation of prompts encouraging students to reflect on patients’ responsiveness to the communication skills used during conversations could possibly enhance the learning effect of the FEEDBK model (Hall C, Peleva E, Vithlani RH, et al., 2020). A communication training model that, similar to the prompt used in our study, is focusing on the intended outcomes by the student is the Agenda-led outcome-based analysis (ALOBA). This is a model that is often used in teaching and learning Silverman’s Communication skills in Medicine and uses the question “what were you trying to achieve?” for the development of learning goals (Kurtz et al., 2017). This prompt could be strengthened by adding a question evaluating the patient’s verbal or non-verbal reaction to evaluate whether the intended goal was achieved during the conversation.

Future research should concentrate on evaluating the impact of different types of prompts on predictive cue use and monitoring accuracy in order to develop a prototype prompt for communication skills training. Additionally, it would be interesting to evaluate the effect of offering prompts more frequently on monitoring accuracy and to analyze the eventual effect on performance by evaluating patients’ satisfaction with trainees’ communication skills.

## Limitations

There are several limitations to be considered in this study. Firstly, since we did not isolate the prompting and feedback procedures during the intervention, we could not test for possible interactions between them. However, since instructors during the Mock OSCE did not know which prompts residents had received and feedback procedures were similar, we think that in case of any effect this was similar for both groups. A second limitation was that the prompt during the intervention was used for self-evaluation whereas the pre- and post-intervention test concerned evaluation of a peer resident’s communication skills. This might have had an influence on the transferability of the prompt -effect. Nevertheless, we classified the quality of self-evaluations and peer-feedback in terms of content and/or style characteristics. Students’ ability to focus on content and style characteristics is generic and transferable to other settings. Using a pre-rated video of a peer allowed us to quantify residents’ monitoring judgements against a standard which was a strength in the study.

A third limitation was that our study looked at single cue use only. Whether learners use single cues to arrive at judgments of learning or whether they integrate multiple cues from various judgment domains remains under debate (Undorf, Sollner, Broder, 2018). Nevertheless, the study by Wagner-Menghin studied combined a, b and c judgements and nonetheless found that JOS c cues were most predictive. For future studies, we suggest eliminating feedback from the experiments as this could potentially introduce a confounding factor. Also, multiple cue-use to arrive at judgments of (dis)satisfaction should considered.

## Conclusion

Prompting students to access predictive cues after a communication OSCE can help them concentrating on relevant cues when evaluating their communication skills. The usefulness of training with actors during an OSCE can be increased if both teachers and learners understand the usefulness of self-reflection and evaluating whether the intended goal during the conversation was reached and to look for signs of success. In communication training, these signs are to be seen almost entirely in the patient. Future research should focus on offering more frequent teaching sessions using predictive prompts over time to evaluate whether this increases the effect on monitoring accuracy in communication skills.
